# Redefining Antimicrobial Resistance in *Acinetobacter baumannii*: A Mechanistic Framework Linking Intracellular Antibiotic Activity to Treatment Failure

**DOI:** 10.3390/ijms27114911

**Published:** 2026-05-29

**Authors:** Ayman Elbehiry, Adil Abalkhail, Saad A. Alotaibi, Eman Marzouk

**Affiliations:** Department of Public Health, College of Applied Medical Sciences, Qassim University, P.O. Box 6666, Buraydah 51452, Saudi Arabiae.marzouk@qu.edu.sa (E.M.)

**Keywords:** *Acinetobacter baumannii*, antibiotic resistance, phenotypic adaptation, biofilm-associated resistance, pharmacokinetics-pharmacodynamics, bacterial persistence

## Abstract

*Acinetobacter baumannii* (*A. baumannii*) is a major cause of multidrug-resistant infections, yet resistance is often interpreted from a gene-centered perspective that does not explain inconsistent treatment outcomes. This narrative review redefines resistance as a treatment-associated phenotype arising from interactions among molecular resistance mechanisms, bacterial physiology, and the infection environment. Unlike previous reviews that examine determinants in isolation, this work integrates efflux, permeability, enzymatic activity, and target modification with phenotypic states such as structured growth, metabolic adaptation, and stress response within a unified framework. Within this framework, bacterial elimination depends on whether antibiotics maintain sufficient target engagement under infection conditions. Antibiotic performance varies with local environment, population diversity, and cellular activity, which explains the gap between laboratory susceptibility results and clinical response. Antimicrobial failure therefore results from the combined effects of impaired drug exposure, adaptive bacterial physiology, and resistance mechanisms within the infection environment. Based on this framework, therapeutic strategies are reconsidered with emphasis on enhancing drug access, modulating cellular behavior, and disrupting population structures that sustain persistence. The review also highlights key gaps, including limited representation of infection environments in experimental models, insufficient resolution of population diversity, and weak integration between pharmacokinetics and bacterial physiology. This framework supports a mechanistic interpretation of resistance and therapeutic response in *A. baumannii*.

## 1. Introduction

*Acinetobacter baumannii* (*A. baumannii*) is a major cause of hospital-acquired infection, particularly in intensive care settings where invasive procedures and prolonged hospitalization are common [1,2,3]. It is frequently associated with ventilator-associated pneumonia, bloodstream infection, and wound infection in critically ill patients. Carbapenem-resistant *A. baumannii* is linked to high mortality, exceeding 20% overall and rising above 40% in bloodstream infection, with similarly high estimates reported in ventilator-associated pneumonia depending on disease severity [4]. Globally, antimicrobial resistance (AMR) contributes to approximately 4.95 million deaths annually, including 1.27 million deaths directly attributable to bacterial resistance [5]. A defining feature of *A. baumannii* is its persistence in hospital environments, where it tolerates desiccation and retains viability on dry surfaces for prolonged periods, sustaining environmental reservoirs and repeated transmission [6,7].

The clinical challenge extends beyond the presence of resistance genes. Carbapenem resistance is commonly mediated by class D β-lactamases, including OXA-23, OXA-24/40, and OXA-58 [8,9]. Additional mechanisms, such as metallo-β-lactamases, reduced membrane permeability, and efflux pump overexpression, also restrict susceptibility. Many of these determinants are carried on mobile genetic elements that facilitate dissemination and persistence in healthcare settings [7,10]. Nonetheless, their presence alone does not fully explain therapeutic failure.

This limitation arises because susceptibility testing evaluates bacterial growth under standardized laboratory conditions, while infection occurs within a dynamic biological environment. Minimum inhibitory concentration measurements do not capture bacterial killing dynamics or survival of less susceptible subpopulations. Non-genetic phenotypes such as tolerance and persistence permit survival without altering measured susceptibility [11,12,13]. Tolerance reduces killing across the bacterial population, whereas persistence reflects transient subpopulations that survive through low-activity physiological states often associated with metabolic suppression. These behaviors help explain treatment failure despite apparent in vitro susceptibility [14].

The hospital environment actively reshapes bacterial responses to antimicrobial exposure by altering both local drug distribution and cellular physiology. Antibiotic distribution varies across tissues, medical devices, and environmental surfaces, generating gradients rather than uniform exposure [15]. Simultaneously, nutrient limitation, oxidative stress, and host defenses alter bacterial physiology. Under these conditions, *A. baumannii* forms biofilms, remodels its envelope, and reduces metabolic activity. Biofilm organization creates diffusion barriers and local gradients in oxygen and nutrients that restrict antibiotic penetration and diminish bacterial killing without altering intrinsic susceptibility [16].

Regulatory networks convert environmental stress signals into coordinated adaptive responses that modify antibiotic susceptibility. The BfmRS two-component system controls surface attachment, biofilm formation, and envelope organization through regulation of the *csu* pili assembly system [17]. The AdeRS system regulates the AdeABC efflux pump and influences cellular drug retention [18]. These pathways regulate gene expression, membrane organization, and transport processes in response to environmental stress.

These findings indicate that antimicrobial efficacy depends on whether antibiotics retain sufficient activity at bacterial targets under infection-specific conditions. Enzymatic degradation, membrane permeability, efflux activity, and bacterial physiology all influence this process. Genetic determinants provide the capacity to reduce antibiotic efficacy. However, environmental conditions and regulatory responses govern how this potential is expressed during infection [19].

Recent reviews summarized the major molecular resistance mechanisms and emerging therapeutic approaches associated with *A. baumannii*, particularly β-lactamases, efflux systems, membrane permeability alterations, and multidrug resistance evolution [20,21]. Nevertheless, less attention has been directed toward how bacterial adaptation and local infection conditions influence antibiotic activity and therapeutic outcome. Where organism-specific evidence remains incomplete, broader bacterial studies are incorporated to provide a mechanistic context for interpreting adaptive responses in *A. baumannii*.

**Figure 1:**
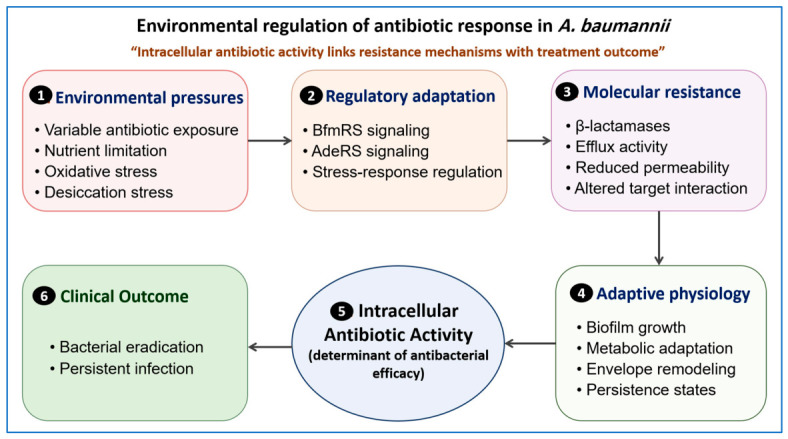
Environmental control of antibiotic response in *A. baumannii*. Conditions within hospital environments influence regulatory systems that shape bacterial physiology and determine antibiotic access to cellular targets, ultimately affecting therapeutic outcome.

This review presents a framework in which antimicrobial failure in *A. baumannii* arises from interactions among resistance determinants, bacterial physiology, and the hospital environment (Figure 1). In addition to summarizing resistance mechanisms, the review examines how antibiotic activity is shaped under infection conditions. Within this model, host-associated conditions influence regulatory systems that coordinate molecular mechanisms with bacterial adaptation, ultimately determining antimicrobial efficacy and clinical outcome.

## 2. Hospital Environment as a Driver of Adaptation in *A. baumannii*

### 2.1. Spatially Heterogeneous Antibiotic Exposure and Biofilm-Associated Survival

Altered pharmacokinetics (PKs) in critically ill patients generate spatially heterogeneous antibiotic exposure that reshapes bacterial survival dynamics across infected tissues [22,23]. Consequently, bacterial populations within the same infection site encounter heterogeneous antimicrobial conditions that promote adaptive physiological responses. Within biofilms, restricted diffusion similarly delays antibiotic penetration and creates spatial gradients in drug availability [15,24]. Cells located at different depths therefore experience distinct exposure conditions. As a result, heterogeneous subpopulations with variable susceptibility to antimicrobial killing can emerge within structured bacterial communities.

These spatial constraints are reinforced by structured biofilm growth on tissues and medical devices. Biofilm-associated communities restrict antibiotic penetration and exhibit greater tolerance than planktonic populations [16]. At the same time, gradients in oxygen and nutrient availability generate physiologically distinct cellular states throughout the biofilm structure. In broader bacterial biofilm systems, inner populations often display reduced activity and diminished responsiveness to antibiotics. Under carbapenem stress, *A. baumannii* increases biofilm formation while maintaining resistance determinants, indicating that antimicrobial pressure reinforces survival-associated phenotypes [25]. Overall, heterogeneous antibiotic exposure and structured biofilm organization reduce bactericidal activity and promote persistence during antimicrobial therapy.

### 2.2. Environmental Persistence, Surface Survival, and Adaptive Transmission

The hospital environment functions as a selective ecological system that continuously promotes physiological adaptation and persistence in *A. baumannii*. *A. baumannii* retains viability on dry hospital surfaces for prolonged periods, particularly within biofilm-associated states that support survival under nutrient-restricted conditions [26,27]. Structural components, including the capsule and outer membrane, enhance tolerance to desiccation and environmental stress, whereas diminished cellular activity during nutrient limitation promotes long-term persistence [28].

Hospital surfaces and medical equipment additionally facilitate repeated transmission through contact with healthcare workers and contaminated devices [27]. As bacterial populations move between abiotic surfaces, medical devices, and host tissues, they encounter fluctuating environmental conditions that require continuous physiological adjustment [3,29,30,31]. These transitions, in turn, generate variation in traits associated with survival, colonization, and dissemination that cannot be explained by resistance determinants alone.

Recent work suggests that persistence under hospital conditions, stress adaptation, and host-associated traits collectively contribute to the long-term success of *A. baumannii* within healthcare systems [32]. Environmental pressures, including nutrient limitation, desiccation stress, and variable antibiotic exposure, therefore select for bacterial populations with distinct physiological characteristics shaped by ecological adaptation rather than gene carriage alone [25,30,31].

**Figure 2:**
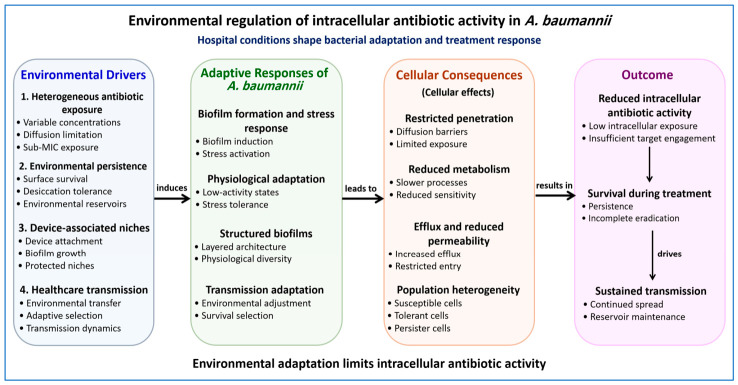
Environmental regulation of antibiotic response in *A. baumannii*. Hospital environments generate spatially and temporally heterogeneous antibiotic exposure while imposing environmental stress, promoting biofilm formation, physiological adaptation, and phenotypic diversity. These responses restrict antibiotic penetration, reduce cellular activity, and alter membrane permeability, thereby reducing antimicrobial efficacy and supporting bacterial survival during treatment.

These environmentally induced adaptive states additionally influence how molecular resistance mechanisms are expressed and function during antimicrobial exposure. Figure 2 and Table 1 summarize the relationships among environmental pressures, bacterial physiology, and antimicrobial response. These environmental pressures influence bacterial behavior in ways that can weaken antimicrobial activity during infection. Therefore, treatment response depends not only on resistance genes but also on how bacterial populations adapt within the hospital environment.

## 3. Molecular Determinants of Resistance in *A. baumannii*

Building upon these environmentally driven adaptive responses, resistance in *A. baumannii* develops through coordinated molecular mechanisms that collectively reduce antibiotic access to bacterial targets. These mechanisms include reduced permeability, active efflux, enzymatic degradation, and target modification [33,34]. They reduce antimicrobial pressure and limit drug–target interaction. Although recent reviews have summarized the major resistance determinants in *A. baumannii*, evidence from broader bacterial systems suggests that antimicrobial efficacy during infection depends on how these mechanisms influence antibiotic access to bacterial targets [33]. Closely related *A. baumannii* isolates can display different resistance phenotypes despite highly similar core genomes. Variation within the ST25 lineage, for example, reflects differences in the accessory genome, including uneven distribution of resistance genes, plasmids, and resistance islands [35]. These mobile genetic elements introduce clusters of resistance determinants that expand the bacterial resistance repertoire.

### 3.1. Genomic Acquisition Defines Resistance Capacity

Horizontal gene acquisition expands resistance capacity by introducing mobile determinants that support multiple complementary resistance mechanisms [36,37,38]. These elements integrate into the genome and form a variable accessory pool that differs across strains. This process allows rapid emergence of multidrug resistance without major genomic change. Resistance determinants also tend to accumulate within specific chromosomal regions that function as integration hotspots and support repeated insertion and recombination events [36]. Mobile genetic elements play a central role in this process. Plasmids mediate inter-strain transfer, while transposons and insertion sequences promote chromosomal integration. Resistance islands can accumulate multiple determinants while still maintaining transfer potential within bacterial lineages [36,37]. Consequently, genomic acquisition provides the molecular foundation for subsequent resistance mechanisms.

### 3.2. Regulatory Activation Determines Functional Expression

Regulatory activation determines whether acquired resistance determinants become functionally integrated into the resistance phenotype. In *A. baumannii*, insertion sequences such as ISAba1 often enhance transcription by providing promoter activity upstream of resistance genes, particularly β-lactamases [39]. Moreover, regulatory systems modulate expression under environmental stress and antimicrobial exposure. Mutations affecting regulatory proteins or promoter regions can increase transcription and produce variable resistance phenotypes despite similar genetic content [40,41].

### 3.3. Enzymatic Degradation Reduces Active Drug

Enzymatic degradation decreases intracellular antibiotic availability by inactivating antimicrobial compounds before target engagement occurs. β-lactamases hydrolyze antibiotics within the periplasm before the drugs reach their targets. This process reduces the amount of active drug available for target engagement. Class D OXA enzymes are central to carbapenem resistance and often function together with other β-lactamases that have overlapping substrate profiles [8,9,42]. Multiple degradative enzymes may coexist within the same isolate. This redundancy broadens substrate coverage and, consequently, stabilizes resistance under different exposure conditions [33,43]. As a result, less of the active antibiotic reaches the cytoplasmic targets.

### 3.4. Efflux Systems Limit Intracellular Accumulation

Efflux systems dynamically lower cellular drug retention by actively exporting antimicrobial compounds after cellular entry. Resistance-nodulation-division (RND) pumps, particularly AdeABC, export antimicrobial compounds across the cell envelope and lower intracellular antibiotic concentration [21,44]. Efflux activity is also influenced by regulatory pathways, since mutations involving systems such as AdeRS or promoter-enhancing insertion elements can induce sustained overexpression and maintain active drug levels below inhibitory thresholds [40]. Efflux therefore acts together with reduced influx mechanisms to limit antimicrobial exposure [39,44].

### 3.5. Target Modification and Restricted Entry

Target modification and restricted cellular entry reduce antibiotic activity through complementary mechanisms that limit drug–target interaction. Mutations in DNA gyrase and topoisomerase IV reduce fluoroquinolone binding and decrease inhibitory activity [45], while membrane alterations restrict antibiotic penetration. Loss or reduced expression of porins such as CarO decreases carbapenem influx [42], thereby reducing the amount of drug reaching the periplasm and cytoplasm. Lipid A modification additionally reduces polymyxin binding to the outer membrane [38]. These structural adaptations influence whether sufficient drug concentrations reach bacterial targets.

### 3.6. Integrated Control of Antibiotic Access and Target Engagement

The resistance phenotype of *A. baumannii* results from coordinated interactions among multiple molecular mechanisms. Genetic acquisition provides resistance determinants, regulatory pathways control their expression, enzymatic activity inactivates antibiotics, efflux systems reduce cellular drug retention, and structural alterations restrict antibiotic entry and target access [36,37,44,46,47]. Available evidence suggests that these mechanisms can act cooperatively: reduced influx limits intracellular penetration, while efflux systems remove residual drug that enters the bacterial cell. Enzymatic degradation additionally decreases active antibiotic availability before target engagement occurs [35,44]. Resistance therefore reflects the combined effect of interconnected molecular processes rather than a single determinant [35,37,46]. Table 2 summarizes the principal mechanisms that regulate antibiotic exposure within bacterial cells.

Table 2 summarizes molecular mechanisms that influence antibiotic availability within bacterial cells, including enzymatic degradation, efflux, reduced permeability, and target modification. However, these mechanisms do not operate independently of bacterial physiology. Their functional impact during infection depends on adaptive cellular states that regulate metabolism, growth behavior, membrane organization, and stress responsiveness under environmental pressure. Resistance therefore develops through the continuous interaction between molecular resistance mechanisms and bacterial physiology rather than from isolated genetic mechanisms alone.

## 4. Phenotypic Adaptation in *A. baumannii*: Determinants of Antibiotic Response

Adaptive physiological states reshape antibiotic susceptibility during infection by altering bacterial growth, metabolism, and cellular responsiveness to antibiotics. These reversible cellular programs alter susceptibility without requiring a genetic change [11,13]. Changes in spatial organization, growth state, membrane structure, and metabolism collectively influence antimicrobial performance during treatment [12]. Thus, bacterial survival during therapy depends not only on molecular resistance determinants but also on the physiological state of the bacterial population under infection conditions.

### 4.1. Biofilm Formation as a Spatially Organized Defense

Biofilm organization spatially restricts antibiotic penetration while simultaneously generating physiologically diverse bacterial subpopulations [50,51]. Accordingly, cells located in deeper biofilm layers encounter lower drug concentrations than surface-associated populations. In *A. baumannii*, biofilm-associated growth is consistently linked to reduced susceptibility and persistence during antimicrobial exposure [52]. Strong biofilm-forming clinical isolates also exhibit greater tolerance to multiple antibiotics than planktonic populations [53,54,55].

Mechanistic insights from broader biofilm studies indicate that biofilm organization generates gradients in oxygen and nutrient availability that produce distinct physiological states throughout the bacterial community [56]. In broader bacterial biofilm systems, inner populations often display reduced growth activity. Similar spatial heterogeneity likely contributes to reduced antimicrobial responsiveness in *A. baumannii* biofilms. Under these conditions, antibiotics targeting active cellular processes become less effective [16]. As a result, metabolic heterogeneity reduces bacterial killing and promotes survival under antimicrobial stress [57,58].

### 4.2. Persistence and Non-Replicative Survival States

Beyond spatial protection within biofilms, bacterial populations can additionally survive antimicrobial exposure through transient low-activity physiological states known as persistence [12,59]. Similar persistence-associated behavior has also been reported in *A. baumannii* [60,61,62]. Evidence from multiple bacterial species suggests that persistence-associated cells survive antimicrobial exposure because many antibiotics require active cellular functions for optimal bactericidal activity. Direct evidence in *A. baumannii* similarly shows that small subpopulations can remain viable after exposure to high antibiotic concentrations despite lacking stable genetic resistance [60,61,62]. Following treatment withdrawal, surviving cells can repopulate the infection site and contribute to recurrent infection [11,12,13].

Available evidence suggests that entry into persistence can occur through stochastic switching or stress-induced physiological responses [11,13]. Environmental stress and antimicrobial exposure increase the frequency of these states and, accordingly, promote prolonged bacterial survival [62]. Therefore, persistence contributes to relapse and chronic infection despite apparent susceptibility during routine testing.

### 4.3. Envelope Remodeling and Permeability Control

In addition to altered growth behavior and persistence, bacterial cells can further reduce antimicrobial susceptibility through structural remodeling of the cell envelope [63]. Changes in outer membrane composition, including altered porin expression and lipid organization, regulate membrane permeability [64]. In *A. baumannii*, reduced expression of porins such as CarO decreases carbapenem influx [65]. Alterations in outer membrane proteins can additionally modify surface characteristics and antibiotic interaction [66]. Modification of membrane-associated structures, including lipopolysaccharide organization, further influences surface properties and antimicrobial susceptibility [67]. These structural adaptations restrict antibiotic penetration and reduce drug penetration under environmental stress conditions.

### 4.4. Metabolic Reprogramming and Reduced Drug Sensitivity

Metabolic reprogramming may modify antibiotic lethality by reducing the cellular activity associated with bactericidal action. In many bacterial systems, environmental stress promotes physiological states associated with reduced growth and altered antimicrobial responsiveness [68]. Across bacterial systems, many antibiotics depend on active cellular processes for optimal killing. Similar metabolic adaptation has also been observed in *A. baumannii* under environmental stress conditions [69,70]. Therefore, reduced metabolic activity diminishes antimicrobial efficacy [71,72], while altered energy availability can additionally affect cellular functions associated with antibiotic susceptibility [72]. These observations suggest that metabolic reprogramming can promote bacterial persistence during antimicrobial exposure and contribute to persistent infection [73].

**Figure 3:**
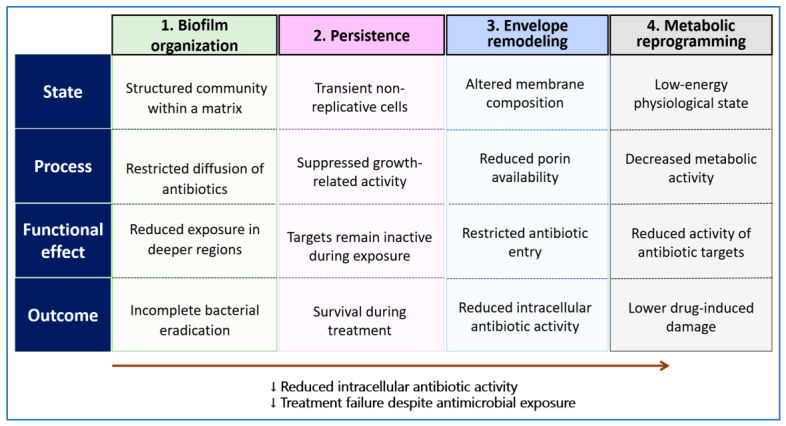
Phenotypic determinants of antibiotic response in *A. baumannii*. Biofilm organization generates spatial gradients that restrict antibiotic penetration. Persistence represents a transient low-activity state associated with tolerance to antimicrobial exposure. Envelope remodeling alters permeability and limits antibiotic entry, even though metabolic reprogramming reduces susceptibility by lowering cellular activity. These adaptive states decrease intracellular antibiotic action and promote survival during antimicrobial treatment. Thus, bacterial survival during therapy reflects the combined influence of adaptive physiology and molecular resistance mechanisms rather than either process alone.

Overall, biofilm organization, persistence, envelope remodeling, and metabolic adaptation weaken antimicrobial killing and promote bacterial survival during therapy. Figure 3 summarizes the major phenotypic determinants influencing antibiotic response in *A. baumannii*.

## 5. Integration of Molecular and Phenotypic Resistance in *A. baumannii*

The adaptive physiological states described above do not function independently of molecular resistance mechanisms. Instead, resistance in *A. baumannii* emerges from coordinated interactions between molecular determinants and bacterial adaptation that jointly regulate antibiotic activity within bacterial cells [19,74]. These interactions dynamically shape antibiotic effectiveness under changing environmental conditions. Molecular resistance mechanisms influence drug access and target interaction, while phenotypic adaptation modifies bacterial physiology, metabolic activity, and cellular susceptibility during antimicrobial exposure.

### 5.1. Coordination Between Efflux Activity and Membrane Barrier Function

Intracellular drug exclusion is achieved through coordinated reduction in membrane permeability and active efflux transport. Reduced porin availability limits membrane penetration, whereas efflux systems remove compounds that enter the bacterial cell [75]. In *A. baumannii*, RND pumps such as AdeABC function together with reduced permeability to maintain reduced intracellular drug levels [76]. Reduced influx lowers the amount of drug entering the bacterial cell, while efflux prevents residual drug accumulation.

Membrane composition influences transporter activity through the incorporation of host-derived fatty acids that alter transporter conformation and affect resistance behavior [77]. Stress–response pathways modify efflux activity and metabolic adaptation, linking environmental conditions with antibiotic response [78]. Through these coordinated effects, cellular drug exposure declines, thereby promoting bacterial persistence during antimicrobial treatment.

### 5.2. Regulatory Networks Linking Stress Responses to Adaptive States

Regulatory networks integrate environmental stress signals with transcriptional programs that coordinate resistance-associated adaptation. Two-component systems and global regulators influence membrane structure, transport activity, and stress tolerance [79]. In *A. baumannii*, these pathways connect environmental cues with changes in efflux activity, surface architecture, and biofilm formation [80]. Therefore, multiple resistance-associated functions can be activated simultaneously under stress conditions.

These regulatory systems also control transitions between planktonic and biofilm-associated states through transcriptional reprogramming and quorum sensing (QS). Transcriptomic analyses demonstrate distinct expression profiles between these states, including increased expression of QS pathways and biofilm-associated structures such as Csu pili and Bap proteins in sessile populations [81]. QS and two-component regulators additionally influence biofilm formation, motility, and resistance-associated traits [82,83]. Through these coordinated responses, environmental stress can alter bacterial physiology and modify antibiotic susceptibility.

### 5.3. Metabolic State as a Central Modulator of Resistance Expression

Cellular metabolic state simultaneously regulates antibiotic lethality and the functional efficiency of resistance mechanisms. Energy-dependent processes such as efflux require active metabolism, and their efficiency declines under low-energy conditions [72]. At the same time, many antibiotics depend on active cellular processes for optimal killing. Because both antibiotic lethality and efflux activity depend on cellular energetics, available evidence within bacterial systems suggests that metabolic state functions as an important determinant of antimicrobial response [84].

Studies across bacterial systems reveal that modifications in respiration and energy flux can influence antibiotic lethality by modulating intracellular damage pathways [85]. Similar metabolic changes have also been demonstrated in *A. baumannii*. Disruption of post-transcriptional regulation through Hfq deletion induces metabolic reprogramming characterized by reduced TCA cycle activity and impaired electron transport [86]. These changes are associated with decreased intracellular ATP and NADH levels together with upregulation of the glyoxylate shunt [86]. Under these conditions, metabolically compromised cells exhibit increased survival during bactericidal exposure. These findings support the concept that metabolic state influences antibiotic susceptibility and persistence, although the full integration of these processes in *A. baumannii* remains incompletely defined.

### 5.4. Integrated Physiological and Molecular Determinants of Resistance

Although direct experimental integration remains limited in *A. baumannii*, available evidence suggests that resistance reflects the combined effects of permeability, transport activity, enzymatic degradation, and metabolic adaptation [13,35,44,74]. These interacting processes likely influence whether antibiotics maintain sufficient activity during infection. Reduced permeability enhances efflux efficiency, while metabolic adaptation influences both antibiotic activity and resistance expression [13].

**Figure 4:**
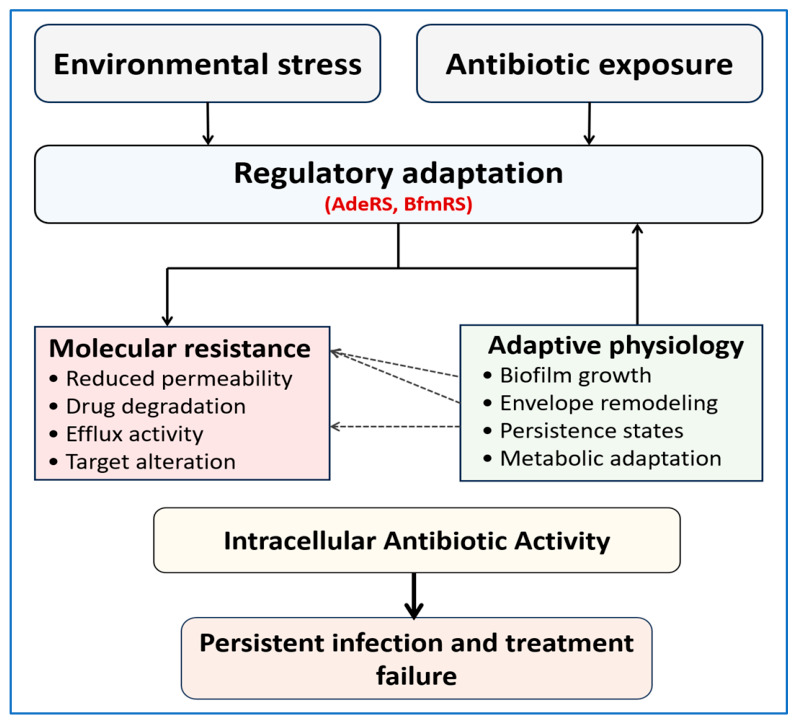
Integrated network regulating antibiotic response in *A. baumannii*. Environmental stress and antibiotic exposure activate regulatory systems that coordinate molecular mechanisms with adaptive physiological states. Reduced permeability, enzymatic degradation, target modification, and efflux function alongside biofilm organization, envelope remodeling, persistence, and metabolic adaptation. These processes regulate antimicrobial action within bacterial cells and determine bacterial survival during antimicrobial exposure. Dashed arrows indicate modulation of molecular mechanisms by phenotypic states.

Integrative analyses show that permeability, efflux activity, enzymatic degradation, and metabolism jointly regulate antibiotic response [19,72,74]. Decreased influx limits intracellular antibiotic entry, whereas enhanced efflux promotes drug removal and enzymatic activity further reduces active drug availability [45]. These mechanisms operate under metabolic constraints in which energy availability influences transport efficiency and contributes to phenotypic heterogeneity [72,84]. Hence, resistance develops through combined effects of molecular resistance mechanisms and adaptive physiological states. Figure 4 illustrates this integrated network regulating cellular antibiotic exposure in *A. baumannii*. These interactions help explain why antimicrobial response can vary even among isolates with similar resistance profiles. Treatment outcome is therefore influenced by dynamic relationships between resistance mechanisms, bacterial physiology, and local infection conditions [13,19,72,74].

## 6. Mechanistic Basis of Antimicrobial Failure in *A. baumannii* Infections

The integrated interactions described in Section 5 become clinically relevant when they impair antimicrobial activity within infection environments. In *A. baumannii*, treatment failure reflects reduced antibacterial efficacy within complex infection sites rather than the presence of resistance determinants alone. Clinical response therefore depends on local drug exposure, bacterial population structure, and cellular physiological state during infection [13,72,87,88].

### 6.1. Spatial Constraints on Antibiotic Distribution

Altered PKs and infection-site barriers generate spatially uneven antibiotic exposure that limits bactericidal activity within infected tissues [22,23,24]. In *A. baumannii*, biofilm-associated growth creates regions with restricted antibiotic penetration [26]. Hence, bacterial subpopulations within the same infection site may experience markedly different antimicrobial conditions. Microenvironmental structure influences local antibiotic concentrations by generating short-range gradients through diffusion, degradation, and uptake within bacterial communities [89].

Experimental studies in bacterial communities demonstrate that resistant subpopulations can reduce surrounding antibiotic concentrations and create localized protection zones for neighboring cells [89]. Although these effects have mainly been demonstrated in experimental microbial systems, similar spatial constraints can contribute to heterogeneous antibiotic exposure in *A. baumannii* infections [90]. Variability in antibiotic exposure also occurs across tissues, medical devices, and environmental reservoirs [90]. Accordingly, portions of the bacterial population are exposed to subtherapeutic concentrations despite appropriate dosing. These uneven exposure conditions likely create selective environments that favor survival of less responsive subpopulations.

### 6.2. Heterogeneous Bacterial Populations Within Infections

Infection-associated bacterial populations develop physiological heterogeneity that produces variable responsiveness to antimicrobial exposure [14,31]. Local environmental variation contributes to this diversity, while antibiotic exposure preferentially eliminates more susceptible fractions of the population. Less responsive cells can therefore survive treatment and sustain infection. This heterogeneity extends beyond metabolic activity alone. Clinical isolates display broad genotypic and phenotypic variation despite conservation of the core genome [91]. Differences in colony morphology, capsule production, and virulence-associated traits indicate coexistence of multiple phenotypes within the same isolate [91].

Comparable variability is also observed during clinical susceptibility testing. Phenotypic heterogeneous resistance refers to subpopulations capable of growth within inhibition zones despite overall susceptibility [92]. This phenotype is associated with prior antibiotic exposure and critical care settings and cannot be consistently detected by standard susceptibility assays. Within bacterial systems, phase variation can expand population diversity through reversible shifts in surface architecture, motility, and stress-associated traits [93]. Comparable adaptive diversification has also been described in *A. baumannii* clinical populations [91].

Overall, these adaptive changes are thought to enhance bacterial persistence under fluctuating environmental conditions [13,91,93]. These forms of phenotypic diversification generate infection-associated populations that respond unevenly to antimicrobial exposure across both spatial and physiological dimensions. Beyond generating heterogeneous bacterial populations, these adaptive states also influence whether bacterial physiology remains compatible with antibiotic-mediated killing.

### 6.3. Mismatch Between Drug Mechanism and Bacterial Physiology

Bactericidal activity depends on adequate interaction between antibiotics and metabolically active cellular targets. In multiple bacterial species, antibiotics targeting DNA replication, protein synthesis, or cell wall synthesis generally require sufficient metabolic activity for optimal killing [72,84]. During infection, however, *A. baumannii* frequently adopts low-activity physiological states in response to nutrient limitation, host pressure, and environmental stress [69,70]. Under these conditions, antibiotic efficacy declines even when drugs successfully reach the infection site.

Antimicrobial performance varies under different experimental conditions. Azithromycin demonstrates weak activity during standard susceptibility testing but becomes active in physiologic media, whereas minocycline exhibits reduced activity under the same conditions [94]. These findings suggest that conventional in vitro testing cannot fully predict in vivo antimicrobial response.

Differences in antimicrobial kinetics additionally influence treatment outcome. Some translation inhibitors act rapidly but transiently, whereas others produce slower yet more sustained inhibition [94]. These differences permit partial bacterial recovery during therapy. Combination approaches help overcome this limitation. The azithromycin–minocycline combination provides both rapid onset and sustained inhibition, improving bacterial suppression in experimental models. These findings support the concept that treatment failure can arise when drug activity becomes mismatched with bacterial physiology during infection [94].

### 6.4. Predictable Failure as a Consequence of Infection-Site Constraints

Antimicrobial failure likely emerges when uneven drug distribution, physiological heterogeneity, and reduced cellular activity collectively prevent sufficient antibiotic activity at the site of infection [19,95]. Restricted penetration lowers local antibiotic concentrations [96], while phenotypically tolerant subgroups survive during treatment [13,97]. Reduced metabolic activity further diminishes the efficacy of antibiotics that depend on active cellular processes [72,98].

Overall, these constraints uncouple measured drug exposure from bacterial killing at the local level, consistent with PK and pharmacodynamic principles [95,96]. Persistence of tolerant and metabolically inactive cells contributes to prolonged infection and therapeutic failure [97,98]. Therefore, clinical failure reflects limitations imposed by the infection environment rather than a single resistance determinant [19]. Interactions among drug distribution, bacterial physiology, and local tissue conditions help explain the frequent discrepancy between laboratory susceptibility results and therapeutic response [99].

Accurate treatment consequently requires strategies that promote tissue penetration, target heterogeneous bacterial populations, and restore susceptibility under infection-specific conditions. Table 3 summarizes the major system-level determinants of antimicrobial failure in *A. baumannii* infections. Accordingly, improving treatment outcomes will require approaches that address both molecular resistance and the adaptive states that support bacterial survival during infection [13,72].

## 7. Strategic Approaches to Overcome Resistance in *A. baumannii*

Successful treatment of *A. baumannii* infections requires restoring antimicrobial activity within complex infection environments [22,23]. Emerging therapeutic reviews increasingly support strategies that combine conventional antimicrobial therapy with approaches targeting biofilm organization, metabolic adaptation, and resistance-associated physiological states [100]. These approaches improve antibiotic access to bacterial targets, restore cellular susceptibility, and disrupt adaptive responses that sustain bacterial survival [72].

### 7.1. Disruption of Biofilm and Structural Barriers

Structured biofilm growth promotes survival by restricting antibiotic penetration and altering bacterial physiological responsiveness. Cells within the extracellular matrix experience reduced penetration and altered physiological states that weaken antibiotic activity [14,15]. The matrix, composed of polysaccharides, proteins, and extracellular DNA, stabilizes this protective structure. Disruption of matrix integrity increases antibiotic access to embedded cells. Enzymatic degradation, interference with quorum sensing, and targeting of surface components such as OmpA and Csu pili reduce structural stability and limit community formation [14,81]. Genetic determinants involved in matrix production, including *pgaABCD*, provide additional intervention points [101].

Experimental evidence supports this approach. Tryptanthrin reduces biofilm formation and downregulates *ompA*, *csuE*, and *bfmR* [102]. p-Coumaric acid disrupts established structures and restores imipenem activity in resistant isolates [103]. Natural compounds such as α-pinene inhibit biofilm formation and downregulate structural and virulence-associated genes in *A. baumannii*, supporting their use as adjunctive strategies [104]. These interventions improve antibiotic penetration. However, sufficient antimicrobial activity is still required for complete bacterial eradication.

### 7.2. Induction of Physiological Susceptibility

Suppressed cellular activity weakens bactericidal efficacy by limiting the active cellular processes required for antibiotic-mediated killing [12,14]. Subpopulations with low metabolic activity therefore survive despite antimicrobial exposure. Evidence from broader bacterial systems suggests that restoring cellular activity can enhance antibiotic response. Experimental studies within bacterial systems show that metabolite supplementation can increase antibiotic uptake and promote bactericidal activity [71]. Regulation of metabolic pathways in biofilm-associated cells can link cellular state to antibiotic susceptibility [105]. Alteration of redox balance and stress–response pathways also increases susceptibility. Studies across bacterial systems show that modulation of respiration and oxidative stress can increase cellular drug uptake and increase antibiotic-induced damage [106,107]. Related stress–response pathways also influence susceptibility patterns in *A. baumannii* [53,108].

### 7.3. Optimization of Drug Delivery and Penetration

Insufficient intracellular drug exposure reduces antibiotic activity. Decreased permeability and active efflux restrict entry and retention, preventing adequate intracellular drug levels despite appropriate dosing [44,65]. Strategies that enhance delivery improve drug access within bacterial cells. Bacteriophage-based approaches show activity against multidrug-resistant isolates and enhance antibiotic efficacy [109]. Clinical observations support this principle. The combination of sulbactam with durlobactam restores activity against carbapenem-resistant strains by inhibiting β-lactamases while maintaining sufficient drug activity at bacterial targets [110,111]. These findings illustrate that improving delivery can restore antibiotic success without altering intrinsic susceptibility.

### 7.4. Coordinated Multi-Target Therapeutic Strategies

Resistance in *A. baumannii* is sustained through interacting molecular and physiological mechanisms that cooperatively weaken antimicrobial killing. Single-target interventions are often insufficient because compensatory mechanisms can maintain bacterial survival [2,14]. Genomic and phenotypic analyses show co-regulation of resistance and virulence traits, supporting integrated intervention strategies [112]. Combination approaches improve efficacy by targeting complementary mechanisms simultaneously. Antibiotic combinations can produce synergistic effects, as demonstrated in colistin-based regimens [113]. Similarly, integration of structural disruption with antimicrobial therapy enhances bacterial killing [103], while phage–antibiotic combinations improve efficacy and reduce emergence of resistance [109].

Multi-component strategies that combine quorum sensing inhibition, metabolic modulation, and antimicrobial therapy disrupt coordinated resistance responses [114,115]. Interference with communication pathways reduces biofilm formation and increases susceptibility [14,116], while metabolic activation enhances drug uptake and response [71,117]. Successful therapy therefore requires coordinated intervention across structural, physiological, and molecular levels to restore antimicrobial response and strengthen therapeutic outcome [118,119].

**Figure 5:**
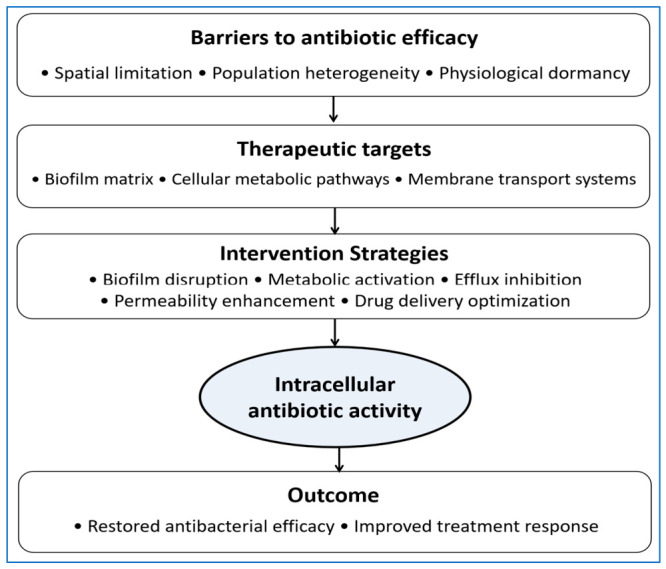
Therapeutic intervention points targeting antimicrobial failure in *A. baumannii*. Antibiotic failure arises from spatial limitation, population diversity, and reduced cellular activity within the infection environment. These constraints define targets including the biofilm matrix, metabolic pathways, and membrane transport systems. Therapeutic strategies act through structural disruption, metabolic activation, modulation of transport, and improved drug delivery. These interventions converge on reinstating antimicrobial activity within infected bacterial populations and improving treatment outcomes.

These therapeutic strategies support a broader interpretation of AMR in which treatment success depends on restoring antibiotic activity within dynamic infection environments instead of targeting isolated resistance determinants alone [13,19,72]. Figure 5 illustrates how targeted strategies act on key biological determinants to restore antimicrobial activity. Table 4 summarizes therapeutic approaches by linking each intervention to its target, mechanism, and functional outcome.

## 8. Integrated Model of Resistance in *A. baumannii*

The preceding sections collectively support a unified interpretation of AMR in which treatment outcome reflects dynamic interactions between bacterial genetics, physiology, and the infection environment.

### 8.1. Dual-Axis Resistance Model

Available evidence supports a model in which resistance in *A. baumannii* develops through dynamic interactions between genetic determinants, bacterial physiology, and infection-site conditions instead of gene carriage alone. Direct evidence in *A. baumannii* shows that isolates can harbor resistance genes yet display variable susceptibility when gene expression or cellular physiology alters their functional impact [120]. Genetic determinants provide the molecular capacity for antibiotic degradation, efflux, and target protection. Yet their contribution to treatment failure varies substantially across infection conditions because phenotypic states such as biofilm-associated growth, metabolic adaptation, and stress tolerance reshape bacterial behavior during antimicrobial exposure [45,120]. Through regulatory pathways that alter transcriptional activity and cellular physiology, environmental signals likely modify how these resistance determinants are expressed in vivo [121].

### 8.2. Context-Dependent Resistance Behavior

Antimicrobial response changes within infection environments because local drug exposure and bacterial physiology continuously interact under dynamic conditions. Variations in growth state, structural organization, and stress tolerance indicate that susceptibility is strongly influenced by local environmental conditions [122]. Environmental heterogeneity can generate bacterial subpopulations with distinct physiological states, including during *A. baumannii* infection [122,123]. Accordingly, treatment response likely reflects ongoing adaptation to local conditions rather than uniform bacterial behavior across the bacterial population. This variability therefore helps explain the frequent discrepancy between standardized susceptibility testing and clinical outcome, where the complexity of infection environments is not fully represented [124].

### 8.3. Implications for Experimental Design and Clinical Interpretation

Conventional susceptibility assays evaluate bacterial growth under uniform laboratory conditions and exclude many factors that influence antimicrobial activity during infection [124]. Hence, these methods may incompletely predict therapeutic response. Experimental systems should therefore incorporate spatial organization, metabolic diversity, and regulatory adaptation to better reflect infection-site conditions [123]. Such models provide a more accurate framework for evaluating antimicrobial performance during infection. Clinical interpretation should also extend beyond categorical susceptibility results. Precise assessment requires determining whether antibiotic exposure remains adequate under the physiological and environmental conditions encountered in vivo [125].

### 8.4. Priority Directions for Future Research

Future advances will require integrating genomic data with bacterial physiology and infection-site conditions to better define resistance behavior during therapy [122]. Approaches capable of resolving population heterogeneity, including single-cell and spatial analyses, will be important for identifying subpopulations that survive antimicrobial exposure [124]. Progress in treatment strategies will also depend on translating mechanistic insight into interventions that target both molecular resistance determinants and adaptive physiological states that sustain persistence during infection [125]. These directions support a broader framework in which AMR is interpreted as a dynamic interaction between bacterial genetics, physiology, and the infection environment rather than as a static property defined solely by resistance genes.

## 9. Conclusions

Antimicrobial failure in *A. baumannii* emerges from dynamic interactions between resistance mechanisms, bacterial physiology, and infection-site conditions rather than from resistance genes alone. Therapeutic outcome depends on whether antibiotics retain sufficient efficacy within the environments where bacterial populations persist. Molecular resistance mechanisms interact with bacterial physiology and local infection pressures to influence antimicrobial performance. Evidence from *A. baumannii,* together with broader bacterial studies, suggests that biofilm organization, metabolic adaptation, and stress-associated physiological states can alter antibiotic penetration and target engagement under infection conditions. This review proposes a unified framework in which resistance is interpreted as a functional and context-dependent behavior rather than a fixed genetic trait. Within this model, antibiotic activity at bacterial targets links molecular mechanisms with clinical outcome and explains the frequent discordance between laboratory susceptibility and therapeutic response. Therefore, accurate treatment of *A. baumannii* infections requires strategies that strengthen drug penetration, target heterogeneous bacterial populations, and restore antibacterial efficacy under infection-specific environments. Some mechanistic concepts discussed in this review are informed by broader bacterial studies because direct experimental evidence in *A. baumannii* remains incomplete for several adaptive and physiological processes. Nonetheless, these comparative observations provide useful mechanistic context for interpreting how environmental stress, metabolic adaptation, and population heterogeneity may influence antimicrobial response during infection. This framework is therefore intended not only to interpret current evidence but also to guide future investigation of resistance behavior under clinically relevant conditions. This integrated perspective integrates organism-specific evidence with mechanistic insights derived from broader bacterial systems to interpret treatment failure under clinically relevant conditions. Incorporation of molecular, physiological, and environmental perspectives provides a stronger framework for understanding antimicrobial failure and developing improved therapeutic approaches against *A. baumannii*.

## Figures and Tables

**Table 1:** Environmental Pressures and Adaptive States Shaping Antibiotic Response in *A. baumannii*.

Environmental Pressure	Specific Condition	Bacterial Physiological State	Impact on Antibiotic Response	Refs.
Variable antibiotic exposure	Uneven concentrations across infection sites in critically ill patients	Stress-adapted cells with altered growth	Reduced bactericidal activity	[22,23]
Diffusion limitation in biofilms	Restricted transport within the biofilm matrix	Spatially heterogeneous subpopulations	Delayed and incomplete antibiotic exposure	[15,24]
Surface desiccation	Dry surfaces and nutrient-poor conditions in hospital environments	Low-activity or persistence-associated states	Reduced susceptibility to antibiotics	[26,27]
Device-associated growth	Biofilm formation on medical devices	Structured communities with diverse physiological states	Impaired antibiotic penetration and survival	[16,29]
Transmission among environments	Movement between surfaces, devices, and hosts within healthcare systems	Continuously adapting populations	Variable response to antibiotic exposure	[3,31]

**Table 2:** Mechanisms Regulating Antibiotic Exposure and Target Access.

Functional Step	Determinants	Mechanism	Effect on Antibiotic Exposure	Refs.
Genomic acquisition	Plasmids; transposons (Tn2006); resistance islands	Horizontal gene transfer and genomic integration	Expands resistance repertoire	[36,37,48]
Regulatory activation	ISAba1; AdeRS mutations	Promoter insertion and transcriptional upregulation	Activates resistance gene expression	[41,47]
Enzymatic degradation	OXA-type β-lactamases; ADC enzymes	Antibiotic hydrolysis	Reduces active drug prior to target interaction	[9,37]
Efflux-mediated extrusion	AdeABC; AdeFGH; AdeIJK	Active transport across cell envelope	Decreases intracellular drug concentration	[44,49]
Reduced influx	Porin loss (CarO)	Decreased membrane permeability	Limits antibiotic entry	[42]
Target modification	gyrA; parC mutations; lipid A modification	Reduced drug–target binding	Lowers antibiotic efficacy	[38,45]
Integrated outcome	Combined mechanisms	Sequential and cooperative effects	Reduced antibiotic access through combined mechanisms	[35,41]

**Table 3:** System-Level Determinants of Antimicrobial Failure in *A. baumannii* Infections.

Clinical Observation	Determinant of Bactericidal Efficacy	Mechanism	Functional Consequence	Refs.
Incomplete clearance despite appropriate dosing	Spatial distribution of antibiotic exposure	Restricted penetration and diffusion barriers within tissues and biofilms	Local antibiotic levels fall below therapeutic thresholds in protected regions	[22,23,24,96]
Recurrence after initial response	Population structure	Coexistence of actively growing and tolerant subpopulations	Surviving cells persist and repopulate after treatment	[13,14,31]
Reduced bactericidal activity	Cellular physiological state	Low metabolic function limits engagement of antibiotic targets	Decreased killing efficiency despite drug presence	[72,84]
Discordance between susceptibility and clinical outcome	Integrated system behavior	Interaction between drug exposure, bacterial state, and local environment	Measured susceptibility does not reflect in situ antimicrobial activity	[19,95]

**Table 4:** Therapeutic strategies targeting resistance determinants in *A. baumannii*.

Strategy	Target	Mechanism of Action	Functional Effect	Refs.
Biofilm disruption	Extracellular matrix and adhesion structures	Degradation of matrix components and inhibition of surface attachment systems	Improves antibiotic penetration and exposes protected cells	[14,15,103]
Quorum sensing inhibition	Cell–cell communication pathways	Interference with signal production or reception controlling collective behavior	Limits coordinated biofilm development and virulence expression	[114,116]
Metabolic activation	Central metabolic pathways	Stimulation of respiration and energy production	Enhances intracellular drug uptake and bactericidal activity	[71,117]
Redox and stress modulation	Oxidative stress and defense systems	Disruption of redox balance and stress adaptation responses	Increases cellular vulnerability to antibiotic-induced damage	[106,107]
Efflux and permeability targeting	Membrane transport systems	Inhibition of efflux pumps or facilitation of drug entry	Increases cellular antibiotic accumulation	[44,65]
Drug delivery enhancement	Tissue and cellular access barriers	Use of delivery systems that improve penetration and localization	Increases antibiotic availability at infection sites	[109]
Enzymatic resistance inhibition	β-lactamase activity	Blockade of antibiotic-degrading enzymes	Preserves activity of β-lactam antibiotics	[110,111]
Combination therapy	Multiple resistance determinants	Concurrent targeting of complementary pathways	Produces synergistic killing and limits adaptation	[113,119]

## Data Availability

No new data were created or analyzed in this study.
